# Pseudo-aneurysm of the hepatic artery after laparoscopic cholecystectomy: A case report

**DOI:** 10.4103/0972-9941.26652

**Published:** 2006-06

**Authors:** G Roche-Nagle, MacEneaney, P Harte

**Affiliations:** Department of Surgery, Mercy University Hospital, Cork, Ireland; * Department of Radiology, Mercy University Hospital, Cork, Ireland

**Keywords:** Gallbladder, hepatic artery, pseudoaneurysm, surgery complication, surgery, therapeutic blockade

## Abstract

Iatrogenic injuries to hepatic artery system may evolve to pseudoaneurysms in the late postoperative period. Although rare, pseudoaneurysms after laparoscopic cholecystectomy can occur, are a serious clinical entity and very difficult to detect.

We present a case of iatrogenic pseudoaneurysm after laparoscopic cholecystectomy. The onset of symptoms occurred 5 days after an uneventful operation. Endovascular coil embolization for a large pseudoaneurysm was unsuccessful and open surgery was conducted. Review of the literature reveals fifty-four more cholecystectomy-related pseudoaneurysms. The site of injury was the right hepatic artery in 61% of the cases and the presenting symptom was hemobilia in two-third of the patients. Embolization was performed in 82% of the cases and surgery undertaken in the remaining 18%.

Knowledge of the condition should result in early diagnosis and thus limit the resultant morbidity. Embolization is the first line of treatment and surgery is reserved for more complex injuries and cases with life-threatening rupture of the aneurysm.

## INTRODUCTION

The majority of the cholecystectomies are now performed laparoscopically and advantages include earlier patient mobilization and hospital discharge. However, the procedure still carries the risks of biliary, vascular and other complications. The incidence of reported vascular complications has been less than that of biliary complications.[1] Vascular injuries during laparoscopic cholecystectomy can occur in an analogous fashion to biliary injuries with potential laceration, transection and occlusion of blood vessels. They are most frequently represented by intraoperative bleeding during dissection of the Calot's triangle, either from the hepatic artery or the portal vein.[2] Although hepatic artery pseudoaneurysm is an exceedingly rare condition, there are several isolated case reports in the international surgical literature. They can occur in the early or late postoperative course and often pose a considerable diagnostic and therapeutic challenge.[2,3] We report the discovery of a pseudoaneurysm of the right hepatic artery, 5 days following laparoscopic cholecystectomy.

## CASE REPORT

A 58-year-old female underwent laparoscopic cholecystectomy and at operation, she had a very inflamed gallbladder, which was firmly embedded into the liver. Oozing from the liver bed was controlled using diathermy. She made a good recovery and was discharged on day 3, postoperatively. Histopathological examination confirmed the presence of a thickened gallbladder with multiple stones.

**Figure 1:**
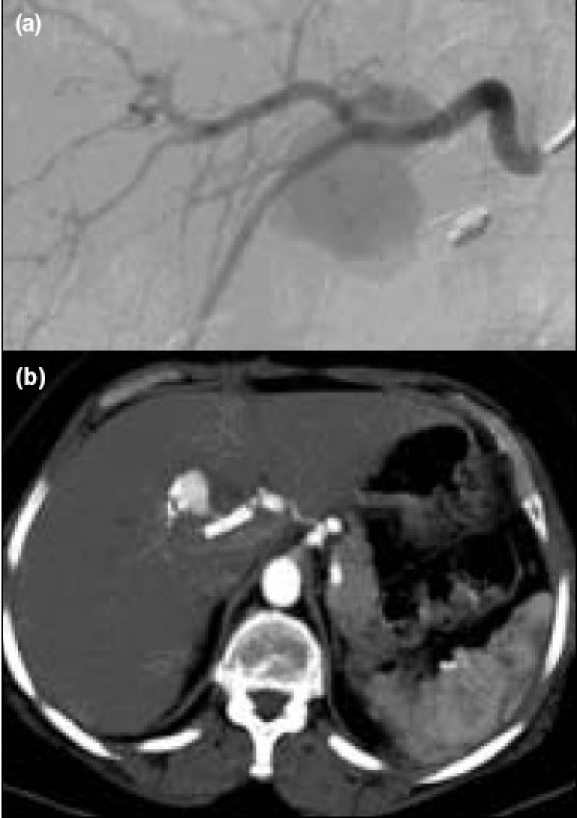
(a) Pseudoaneurysm arising from a branch of the right hepatic artery, in the area above a group of surgical clips, (b) Progressive growth of pseudoaneurysm despite covered arterial stent deployed in the right hepatic artery over the mouth of the feeding artery

Five days after the original surgery, the patient was readmitted to the hospital following an episode of collapse at home. On admission, she appeared unwell with a fever of 38.3°C and a tachycardia of 100/min. Her abdomen was diffusely tender with maximal guarding over the right upper quadrant, but no peritoneal signs were elicited. Plain film of the abdomen showed no pathology, but her hemoglobin was 6.1 g/dl and there was a slight increase in liver enzymes (aspartate aminotransferase [AST], 117 UI/L; alanine aminotransferase [ALT], 108 UI/L). IV fluid resuscitation, blood transfusion and antibiotic treatment was initiated. Computed tomography (CAT) of the abdomen was obtained and revealed some intra-peritoneal blood. After resuscitation and transfusion to a hemoglobin of 10 g/dl, the patient was very well for the following three days. However, on day 4, the patient suffered further abdominal pain and the hemoglobin dropped to 7.3 g/dl. Repeat CT of abdomen was acquired, which again showed intra-abdominal blood and a 3-cm-diameter contrast-enhancing structure in the gallbladder fossa, above the surgical clips. An arteriogram was performed with selective coeliac and superior mesenteric cannulation. This revealed a pseudoaneurysm arising from a branch of the right hepatic artery, in the area above a group of surgical clips [Figure 1a]. The catheter was advanced into the right hepatic artery and multiple coils (Nestor) were deposited into the pseudoaneurysm. In addition, a covered arterial stent was deployed in the right hepatic artery over the mouth of the feeding artery.

**Figure 2:**
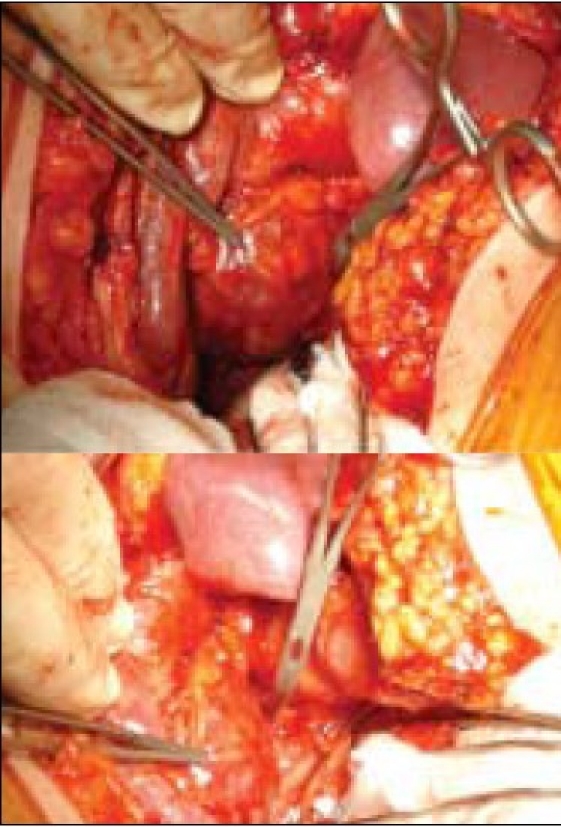
Large pseudoaneurysm in the gallbladder fossa at operation

The patient tolerated the procedure well, maintained hemoglobin levels over the following days and was discharged from hospital. An initial post- procedure CT showed partial thrombosis of the aneurysm. Serial CTs documented a progressive growth over the following two months [Figure 1b]. An exploratory laparotomy via a Kocher's incision was performed and this revealed a large pseudoaneurysm in the gallbladder fossa [Figure 2]. The right hepatic artery was controlled at its origin. The pseudoaneurysm was opened, revealing a feeding artery. This vessel was sutured ligated and the sac closed. The patient made an uneventful recovery.

## DISCUSSION

Laparoscopic cholecystectomy is reportedly associated with an increased incidence of biliary and vascular injuries. Specifically, biliary injuries are reported in 0.2–1% of procedures with a 10-fold increase as compared to open surgery, whereas vascular injuries occur in 0.25–0.5% of procedures.[3] These complications are associated with major morbidity after laparoscopic cholecystectomy.[2] Most vascular injuries consist of intraoperative bleeding from the cystic artery stump, the right hepatic artery or (less frequently) the portal vein. Hepatic artery pseudoaneurysms are rare complications of laparoscopic cholecystectomy, variably occurring in the early or late postoperative period. Other causes include abdominal trauma, liver surgery and less commonly, percutaneous interventional procedures involving the liver, such as biopsy or biliary stent placement.

Patients with hepatic artery pseudoaneurysm present with abdominal pain or discomfort, GI bleeding and alterations in the liver function test (LFT) profile.[2,4] An abdominal mass with bruit may be detected on physical examination. Rupture into the peritoneal cavity, portal vein or pancreatic pseudocyst can also occur.[2] However, the classical clinical triad described by Quinke in 1871[5] - i.e., right upper quadrant pain, jaundice and hemobilia - and has been reported only in 20–30% of patients with laparoscopic cholecystectomy-related hepatic artery pseudoaneurysm.[1]

The diagnosis of pseudoaneurysm is difficult unless the condition is suspected. Ultrasound may reveal a hypoechoic, pulsatile mass within the liver, with bi-directional flow on doppler. Although contrast- enhanced CT will not demonstrate the erosive vascular changes as seen on arteriograms, it may demonstrate hemorrhage or pseudoaneurysm formation, which may be missed on ERCP and angiogram because of the intermittent character of the bleeding episode. The most reliable diagnostic test is selective celiac and SMA angiography. The angiographic feature most frequently associated with hemobilia originating in liver and bile ducts, is pseudoaneurysm.

The exact mechanisms responsible for post- cholecystectomy hepatic artery pseudoaneurysm are not well understood. Contributing factors include vascular erosion secondary to clip encroachment, direct lesion of the vascular wall during surgery and electric current diffusion through clips placed in close proximity to the vascular pedicle.[4] Some authors have suggested that pseudoaneurysms may result from postoperative bleeding from the cystic artery stump, with subsequent inflammation and erosion of the blood clot into the vessel wall. In our case, we hypothesise that the use of diathermy damaged the wall of an artery running along the liver bed, with subsequent pseudoaneurysm formation. Undoubtedly, the wide spectrum of potential mechanisms accounts for the different modes and time of presentation of hepatic artery pseudoaneurysm reported in the international literature.

In conclusion, pseudoaneurysm following laparoscopic cholecystectomy is a recognised and potentially fatal complication. A high index of suspicion is warranted in the presence of hemobila, LFT alterations and abdominal pain, given the poor specificity of clinical symptoms. However, these lesions are amenable to endoscopic and angiographic management, with low related morbidity and favourable long-term results.
